# Open standard is the new open source

**DOI:** 10.7189/jogh.12.03042

**Published:** 2022-07-06

**Authors:** Chris Macek, Brad Cunningham, Nicolas Boillot

**Affiliations:** SystemOne, Northampton, Massachusetts, USA

Global health has been historically committed to adopting open-source software in health applications globally. This is due to a well-intentioned commitment to capacity-building and the belief that open-source systems enable in-country capacity to take over technology implementations without being locked into specific vendors or suppliers. As one 2010 article put it, open-source systems “encourage collaboration, transparency and efficiency, while investing money into local capacity-building instead of software licenses” [1]. However, the shift in stance to requiring open standards over open source is a promising new opportunity to accelerate digital interventions in Global Health while maintaining the historical investment in existing systems.

USAID’s Digital Strategy 2020-2024, calls for the use of “open standards, open data, open source and open innovation” [2]. Often “Open Source” systems are thought to be “free”, yet “open-source” does not always signify “free of cost” or “free of intellectual-property rights.”

According to IBM’s Cloud Computing News, “open source code is created to be freely available, and most licenses allow for the redistribution and modification of the code by anyone, anywhere, with attribution” [3].

For global health, using open-source technology is intended to limit “vendor lock-in” and promote the development of in-country capacity and resources to eventually take over ownership, maintenance, and upgrades for the technology. Unfortunately, reality has not played out that way. Open-source projects have been hard to develop (imagine redeveloping a car from scratch) as ministries of health usually have little to no experience with software development departments and teams. There is also a scarcity of talented developers in low- and middle-income countries [4,5], as they are quickly absorbed into the private sector by large financial and technology firms [6,7], making it even more difficult for a ministry of health to create their own software development arms.

Consequently, many open-source projects have become unsustainable for host countries.

Of course, some well-known open-source projects have been sustained over the long term, such as Linux, OpenMRS and DHIS2. These are usually sustained by a champion for-profit organization that built a suite of proprietary products on top of the open-source core product. In Linux’s case, most software patches and upgrades are contributed by private companies, such as Red Hat and IBM [8]. In global health, the most well-known open-source project, OpenMRS, has been sustained by a rare coalition of supporters comprising some of the original founders, Partners in Health, the Rockefeller Foundation and the World Health Organization, and a host of private companies [9]. OpenMRS gives the impression that open-source projects often work like this, but most fail because they cannot establish a committed and competent global community of developers to fix, maintain and upgrade the technology or even afford to attract such a community in the first place.

**Figure:**
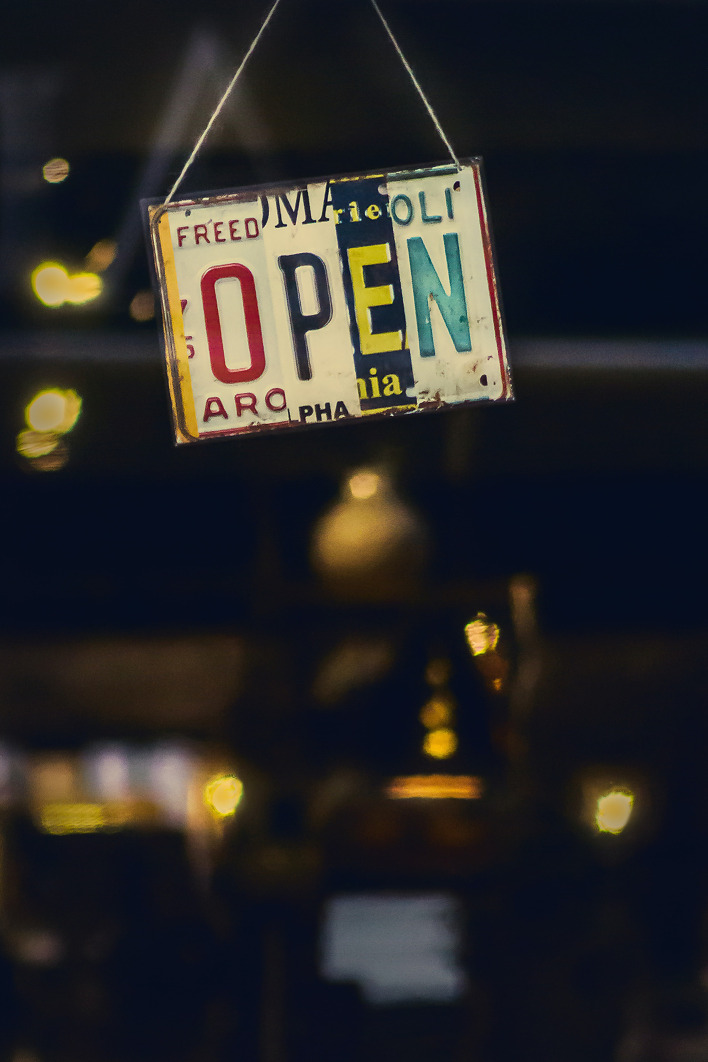
Photo: Photo by James Sutton on Unsplash.

Open standards are different. The aforementioned IBM article defines these as standards that are “freely available for adoption, implementation and updates.” The shift in global health to pen standards over open source signifies that the historical model has not achieved the desired outcomes and that the continued effort to expand and promote private-public partnerships is gaining traction [10-14]. The private sector can (and must) have a significant role to play in solving health care’s most complex issues. Open standards are the gateway to unlocking previously inaccessible private-sector innovations. They not only allow existing historical health systems to connect to new, advanced technology platforms, but provide a mechanism for the private sector to reach a previously unreachable audience locked in siloed, vertical health systems.

The point of open standards is that they are already in use, often ubiquitous, and that it is easier to find engineers who understand them, no matter where the project is located. An open-standards project will:

- **Be faster to fund and develop.** It will also be easier for those specifying and evaluating the project and nurturing in-country skills required to work with it.
- **Avoid vendor lock-in.** It will be easier for multiple vendors to work within the same project, pulling what they need out of it or building modules to interact with it as needs and technology evolve.
- **Benefit vendors.** The choice of open standards will most likely cut across a multitude of projects, allowing the vendor to keep staff up to date and ultimately reduce their costs.
- **Be sustainable for implementing countries.** Developers will want to train on widely shared technology, rather than learning skills that only apply to a single project.
- **Leverage existing historical systems**. Since open standards are intended to promote data sharing between systems, there will be no need to replace historical siloed vertical health applications when these systems can be integrated into more comprehensive platforms without needing to replace them.

## FOSTERING INNOVATION AND PROVIDING OPTIONS

Focusing on open standards rather than open source will foster more innovation and provide more options to global health aid recipients. Requiring open source is often interpreted as a requirement to develop from scratch and give the code to the implementing country. This severely limits the number of organizations that can deliver on an RFP. As previously mentioned, it also limits the possibility of future development by the host country.

Conversely, requiring open standards enables numerous vendors to get in the game, as they do not have to invent solutions from scratch and can focus instead on innovating new value out of existing, open standard-based technologies. They will be better able to budget and more likely to deliver a product that can be maintained over the long term.

The funding world has created a false equivalence between ownership and sustainability, which is why open source has been in such high demand. But the result has been a massive number of technologies that fail as soon as implementing contracts end. 98% of open-source projects on GitHub only have modifications in their first year and are never touched again [15]. Because these projects are software, the failed technologies are not visible to the naked eye in the way one can see hardware graveyards in numerous developing countries, so the problem is hidden. But we can and must do better. The technologies are available. The engineers are available. Focusing on open standards instead of open source will unleash a wave of creativity innovating new, sustainable technologies for the future of global health.
